# Failure of Major Upper Extremity Replantation Due to COVID-19-Related Arterial Thrombosis

**DOI:** 10.7759/cureus.14721

**Published:** 2021-04-27

**Authors:** Ali Eray Günay, Mehmet Çavuş, Kürşat Tuğrul Okur, Murat Kahraman, İbrahim Altun

**Affiliations:** 1 Orthopedics and Traumatology, Kayseri City Hospital, Kayseri, TUR; 2 Hand Surgery, Kayseri City Hospital, Kayseri, TUR

**Keywords:** upper extremity trauma, covid 19, upper extremity thrombosis, limb replantation, traumatic amputation

## Abstract

Coronavirus disease of 2019 (COVID-19), caused by the new severe acute respiratory syndrome coronavirus 2 (SARS-CoV-2) that emerged in Wuhan, was declared a pandemic by the World Health Organization. COVID-19 has many different clinical manifestations. One of them is arterial hypercoagulopathy. Although its mechanism is not fully explained, acute thrombosis and thromboembolism can be seen in patients. In this study, we present a case who was amputated due to the development of arterial thrombosis on the 10th day following infection with coronavirus, despite successful replantation after traumatic above-elbow amputation. After replantation on the seventh day, it was learned that the patient’s husband was positive for COVID-19 and had come to visit the patient. For this reason, we performed reverse transcription polymerase chain reaction (RT-PCR) to confirm the patient’s COVID-19 status. We found that the patient, who was asymptomatic, was positive by RT-PCR for COVID-19. On the 10th day after the operation, it was observed that the blood circulation of the replanted extremity was impaired, although it had been perfect until that day. Emergency embolectomy and vascular reanastomosis were planned for the patient. Although we generally observe thrombosis at an end-to-end anastomosis site, massive axillary arterial thrombosis was detected at the proximal end of the vascular anastomosis. Upon development of tachycardia, hypotension, and metabolic acidosis after embolectomy and vascular reanastomosis, the decision was made to amputate the replanted limb to reduce the risk of life-threatening complications. To our knowledge, this is the first such COVID-19-related complication on upper extremity replantation in the literature.

## Introduction

Approximately six million people visit the ER annually due to upper extremity injuries [1]. The most devastating form of these injuries is upper limb amputations, which are often caused by motor vehicle and industrial accidents [1]. Although the method of treatment is decided according to many variable factors, such as that of mechanism injury, patient preference, duration of ischemia, surgeon's preference, or comorbid conditions, there is still a debate about the indication for above-elbow replantation because of its unusual occurrence [1,2].

Post-replantation follow-up is as important as the replantation process [2]. Metabolites in long-term ischemic tissue enter the venous system after replantation, which can lead to additional problems on a scale that can cause hyperkalemia, myoglobinuria, acute renal failure, and death [3-5]. For this reason, deciding on replantation and following a replanted patient is as difficult a process as the procedure itself [3-5].

A perfect supply of arterial and venous flow is essential for the survival of the replanted limb [5-7]. A major cause of early loss of a replanted limb is deficiencies in vascular anastomosis [5-7]. The replanted limb may be lost due to arterial or venous insufficiency, especially in the postoperative first week [6]. In case of failure, vascular anastomoses can be renewed [6,7].

Coronavirus disease of 2019 (COVID-19), caused by the new severe acute respiratory syndrome coronavirus 2 (SARS-CoV-2) that emerged in Wuhan, was declared a pandemic by the World Health Organization [8]. Clinically, COVID-19 varies among individuals, ranging from asymptomatic infection to severe respiratory failure [8]. Nasal swab, tracheal aspirate, or bronchoalveolar lavage (BAL) is used for reverse transcription polymerase chain reaction (RT-PCR) to diagnose COVID-19 [8]. Common clinical patterns of COVID-19 are dyspnea, severe interstitial pneumonia, acute respiratory distress syndrome, and multiorgan dysfunction [8]. One of them is arterial hypercoagulopathy [8-9]. Although its mechanism is not fully explained, acute thrombosis and thromboembolism can be seen in patients [9].

In this study, we present a case who was amputated due to the development of arterial thrombosis on the 10th day following infection with COVID-19, despite successful replantation after traumatic above-elbow amputation. To our knowledge, this is the first such COVID-19-related complication on upper extremity replantation in the literature.

## Case presentation

**Figure 1:**
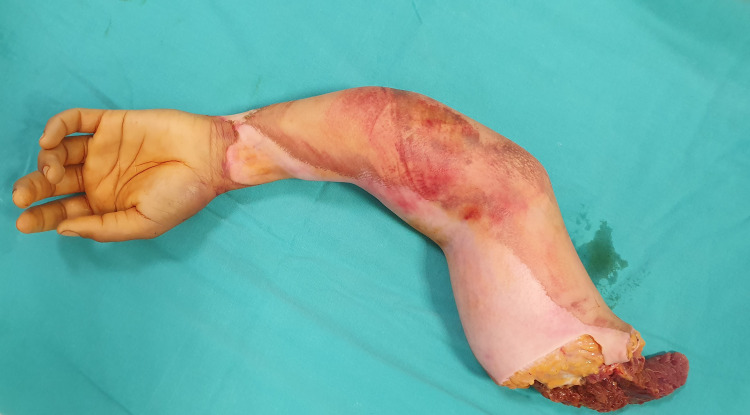
Patient’s physical findings after injury.

**Figure 2:**
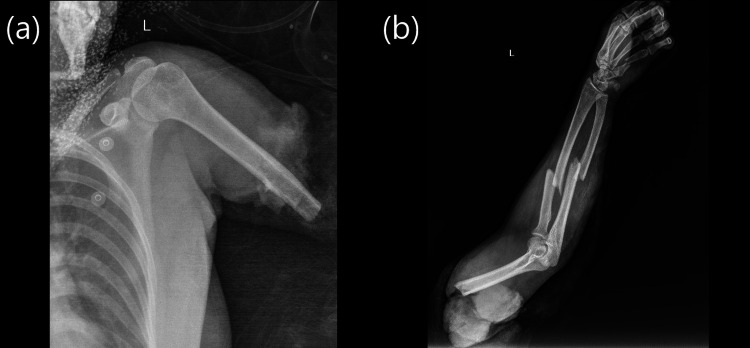
X-ray images of the patient’s left upper extremity (a, b).

A 19-year-old female agricultural worker presented to the ER with a traumatic left arm total transhumeral amputation resulting from an agricultural machine injury (Figure 1). In the first hour after the injury, the amputated part was brought in a plastic container, wrapped in gases, and filled with ice batteries. In addition, the patient had a forearm double bone fracture and a fifth metacarpal fracture of the amputated arm (Figure 2). Mangled extremity severity score (MESS) was calculated as seven.

**Figure 3:**
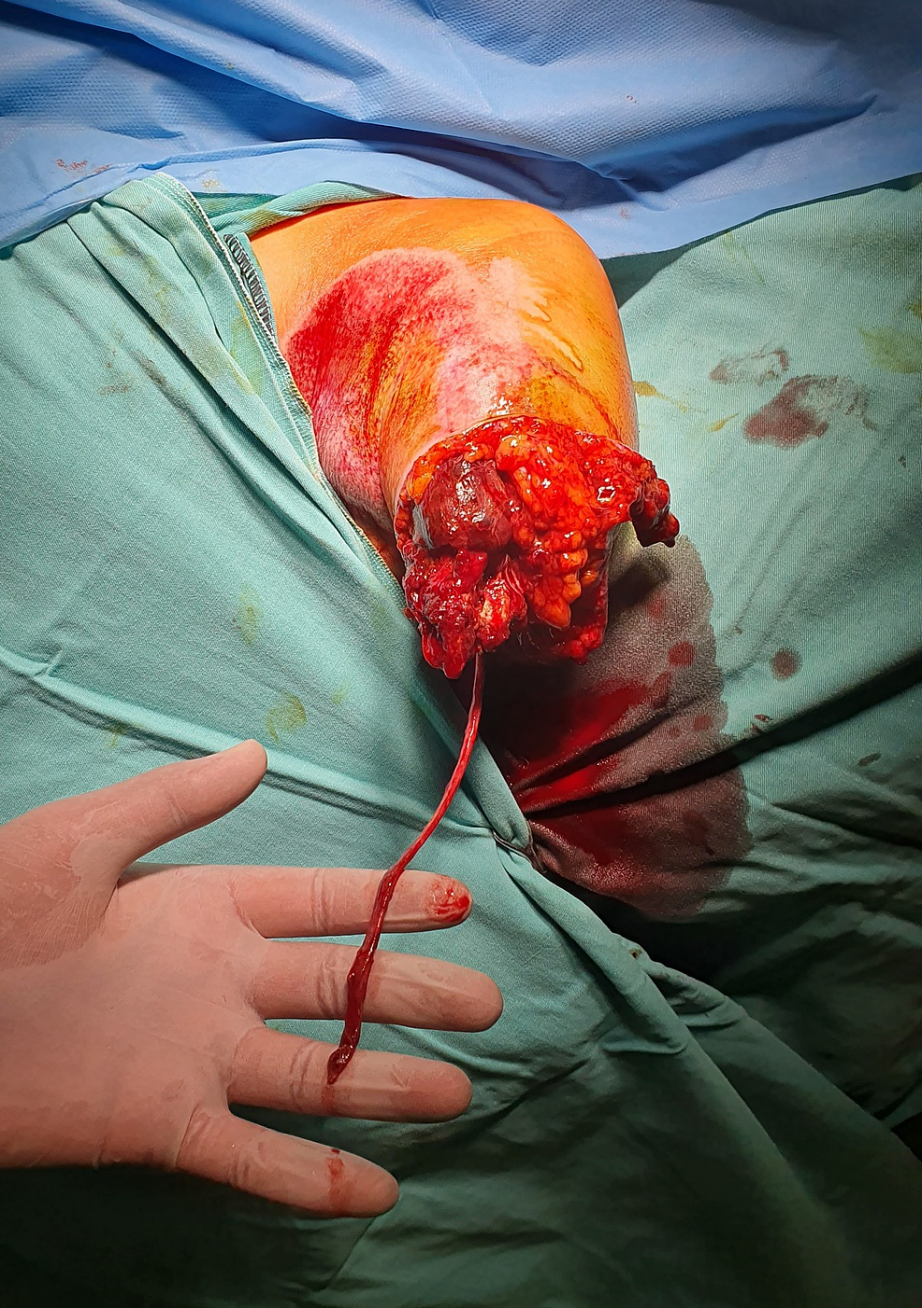
Ulnar nerve avulsed from the wrist level.

On examination of the patient in the operating room, it was found that the ulnar nerve was avulsed from the wrist level and the median nerve from the brachial plexus level, and there were no reparable nerve endings. The radial nerve, brachial artery laceration was established at the amputation level (Figure 3).

**Figure 4:**
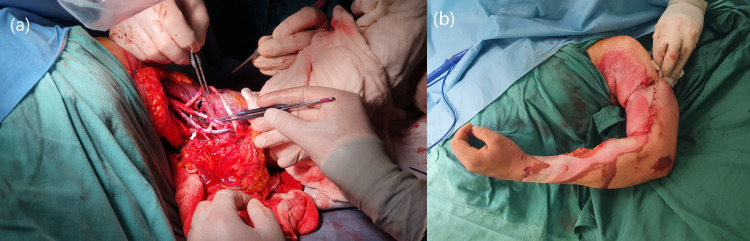
(a) Intraoperative images of brachial arterial (black arrow) and brachial vein end-to-end anastomosis (white arrow) at the amputation level. (b) Patient’s physical findings after replantation.

The patient was operated on under general anesthesia with supraclavicular nerve blockage. Before replantation, the forearm fracture was fixed with a titanium elastic nail; the metacarpal fracture was not fixed. The arm stump was debrided and irrigated, the humerus was shortened by 3 cm, and the amputated limb was fixed with a low-contact dynamic compression plate. End-to-end anastomosis of the brachial artery and vein was performed at the amputation level. Arterial blood flow was provided to the extremity at the fourth hour following the injury. The proximal radial nerve stump was attached to the median nerve that avulsed from the brachial plexus and the ulnar nerve that avulsed from the wrist level to the distal radial nerve stump. After the bleeding was controlled, fasciotomies were applied on the arm and forearm. Following circulation control and dressing, a splint was applied (Figure 4).

Dextran-40 and low-molecular-weight heparin were administered to the patient. Ampicillin sulbactam was given four times a day. The dressing was changed every 48 hours in the operating room. On the seventh day, it was learned that the patient’s husband was positive for COVID-19 and had come to visit the patient. For this reason, we performed RT-PCR to confirm the patient’s COVID-19 status. We found that the patient, who was asymptomatic, was positive by RT-PCR for COVID-19.

**Figure 5:**
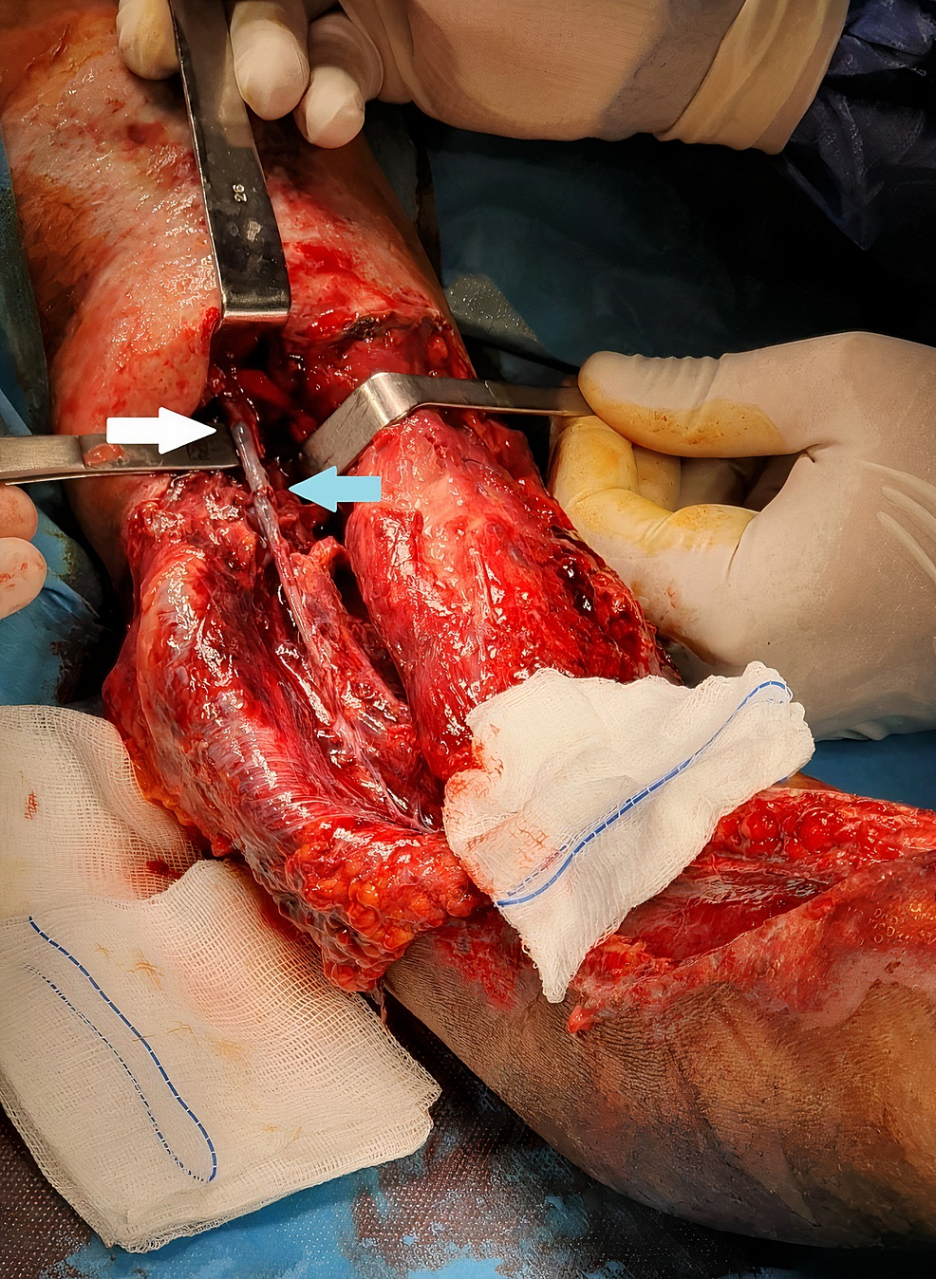
Massive axillary artery thrombosis (white arrow) is detected at the proximal end-to-end anastomosis site (blue arrow).

On the 10th day, it was observed that the blood circulation of the replanted extremity was impaired, although it had been perfect until that day. Emergency embolectomy and vascular reanastomosis were planned for the patient. Although we generally observe thrombosis at an end-to-end anastomosis site, massive axillary arterial thrombosis was detected at the proximal end of the vascular anastomosis (Figure 5). Upon development of tachycardia, hypotension, and metabolic acidosis after embolectomy and vascular reanastomosis, the decision was made to amputate the replanted limb to reduce the risk of life-threatening complications.

## Discussion

Early complications of major upper extremity replantation include arterial and/or venous collapse or infection [6,7]. As a result of arterial thrombosis, a decrease in capillary circulation, decrease in extremity temperature, and decreased or absence of pulse sensation may occur within minutes after replantation and can be further observed for a period of up to eight days [6]. In our case, the patient's capillary circulation was perfect for nine days postoperatively and replanted extremity oxygen saturation was measured as 99%-100%. On the 10th day, peripheral capillary circulation was impaired, the distal pulse could not be taken, the coldness was observed in the replanted extremity, and she underwent emergency surgery.

COVID-19, which causes “cytokine storm” and endothelial damage by increasing the expression of proinflammatory cytokines such as interleukin (IL)-6 and tumor necrosis factor-alpha (TNF-α), along with inflammatory chemokines, predisposes to deep vein thromboembolism or arterial thrombosis [9,10]. Additionally, activation of angiotensin-converting enzyme-2 receptor and macrophage in the vascular endothelium can lead to this condition [11].

After the pandemic was declared, it was observed that the frequency of acute limb ischemia in the Lombardia region increased significantly compared to last year [12]. In addition, too many COVID-19-related venous thromboembolisms in the early period, stroke, acute coronary syndrome, or extremity gangrenes due to arterial thrombosis have also been reported [13-15]. Hanif et al. [11] and Shao et al. [16] have presented acute upper limb ischemia due to COVID-19 and stated that arterial thrombosis can be the first symptom of patients in COVID-19. Although it has been reported to have a 3.7% incidence in intensive care patients, the true incidence of arterial thrombosis in both upper and lower extremities is unknown. Therefore, anticoagulant and antiaggregant drugs are used in the treatment of COVID-19 [9-12]. After COVID-19 infection, the risk of increased thrombosis in the proximal vessels is reported by Goldman et al. [13]. Massive axillary arterial thrombosis was detected in our case report. While the case mentioned above was followed without any problem, arterial thrombosis developing after COVID-19 infection suggests that this situation is due to viral infection.

## Conclusions

Post-replantation care is as important as the surgical procedure. Although it has not been clearly demonstrated, COVID-19 can predispose patients to arterial thrombosis and may affect replantation success. For this reason, protection of vascular repair or replanted cases from COVID-19 is important to safeguard patients, and COVID-19 before replantation can be vital in making treatment decisions.
